# Progression of Nasopharyngeal Pleomorphic Adenoma to Carcinoma Ex Pleomorphic Adenoma With Metastases: A Case Report

**DOI:** 10.7759/cureus.97776

**Published:** 2025-11-25

**Authors:** Krystsina Zhukovich, Alisher Tashbayev, Vladimir Osipov

**Affiliations:** 1 Pathology, AlphaMed Medical Center, Minsk, BLR; 2 Radiology, Whangarei Hospital, Whangarei, NZL; 3 Pathology, Whangarei Hospital, Whangarei, NZL

**Keywords:** carcinoma ex pleomorphic adenoma, fish, her2-positive, metastases, nasopharynx, plag1, pleomorphic adenoma, salivary gland tumours

## Abstract

Pleomorphic adenoma (PA) is the most common benign salivary gland tumour, typically arising in the parotid gland, with minor salivary gland involvement being less frequent and nasopharyngeal localization exceedingly rare. Malignant transformation into carcinoma ex pleomorphic adenoma (CXPA) occurs rarely, but metastatic disease is exceptionally uncommon. To date, no cases of nasopharyngeal PA transforming into CXPA with both nodal and distant metastases have been reported.

We describe a 60-year-old man who presented with progressive facial pain, paraesthesia, weight loss, and cervical lymphadenopathy. Imaging revealed a destructive maxillary and nasopharyngeal mass with perineural spread, nodal disease, and disseminated metastases to the lung, liver, and bone. Initial nasopharyngeal biopsy demonstrated only benign PA with cartilaginous stroma and epithelial elements, while lymph node biopsy revealed poorly differentiated carcinoma with positivity for broad-spectrum cytokeratin, GATA3, HER2 (3+ membranous), androgen receptor, but negative for p40 and p16. Fluorescence in situ hybridization (FISH) demonstrated PLAG1 gene rearrangements in both the benign nasopharyngeal lesion and the metastatic carcinoma, establishing the diagnosis of CXPA arising from a nasopharyngeal PA.

This case underscores several diagnostic challenges: (1) histology from the primary lesion initially suggested a benign tumour, discordant with aggressive radiological features; (2) ancillary molecular testing with PLAG1 FISH was critical in linking the primary PA with metastatic carcinoma; and (3) HER2 overexpression and androgen receptor positivity highlighted potential therapeutic targets, though the rapid disease course precluded treatment. Correlation of imaging, histology, cytology, and molecular studies is crucial, particularly when biopsy findings appear discordant with clinical behaviour.

## Introduction

Salivary gland tumours are rare among head and neck neoplasms. Pleomorphic adenoma (PA), also known as a benign mixed tumour, is the most common primary salivary gland epithelial neoplasm. Histologically, it is a triphasic neoplasm characterized by a mixture of epithelial and myoepithelial cells embedded within a chondromyxoid stromal background. Although hormones and radiation exposure are suggested as risk factors, the etiology remains unconfirmed [1]. Most commonly, PA arises in the parotid gland, followed by the submandibular gland [1]. Tumours originating from the minor salivary glands are relatively uncommon, accounting for approximately 10-25% of all salivary gland tumours [2]. Among these, PA - also referred to as a benign mixed tumour - represents the most frequent histological subtype, constituting approximately 65% of all salivary gland tumours and nearly 40% of those arising from minor salivary glands [3]. If it originates from minor salivary glands, the palate is the most frequent site, but it may also occur in the trachea, larynx, sinuses, upper lip, or cheek [3]. Its occurrence in the nasopharynx is rare, with only 22 cases reported. Heterotopic minor salivary glands are the most likely source of PAs in unusual sites, such as the nasal cavity, pharynx, jaw, or lymph nodes. Patients with minor salivary gland PA typically present in the fourth to sixth decades of life [4], with a slight female predominance [5]. Clinical presentation may include unilateral nasal obstruction, epistaxis, nasal swelling, an intranasal mass, and mucopurulent rhinorrhea [6].

The malignant transformation rate of PA is not precisely established, as many carcinomas ex pleomorphic adenomas (CXPAs) present without prior clinical history of PA. CXPA histological diagnosis requires microscopic confirmation of both benign (PA) and malignant components. The latter could have histomorphology of salivary duct carcinoma, myoepithelial carcinoma, or epithelial myoepithelial carcinoma. Progression to malignancy in recurrent PAs is very infrequent (the rate is around 3%) [1]. Known risk factors include older age, recurrent disease, radiation exposure, submandibular location, large tumour size, prominent hyalinization, and high mitotic activity. Clinically, CXPA may present with pain, ulceration, or nerve palsy.

## Case presentation

A 60-year-old man presented in January 2025 with right upper jaw pain, facial numbness, and weight loss of eight kilograms over four weeks and cervical lymphadenopathy, progressively worsening over six weeks. In February 2025, he had attended the emergency department with a severe right-sided headache, oral pain, facial paraesthesia, fatigue, and further weight loss. He had previously been treated with antibiotics by a dentist for presumed dental infection. He reported photophobia, and his partner noted confusion.

**Figure 1:**
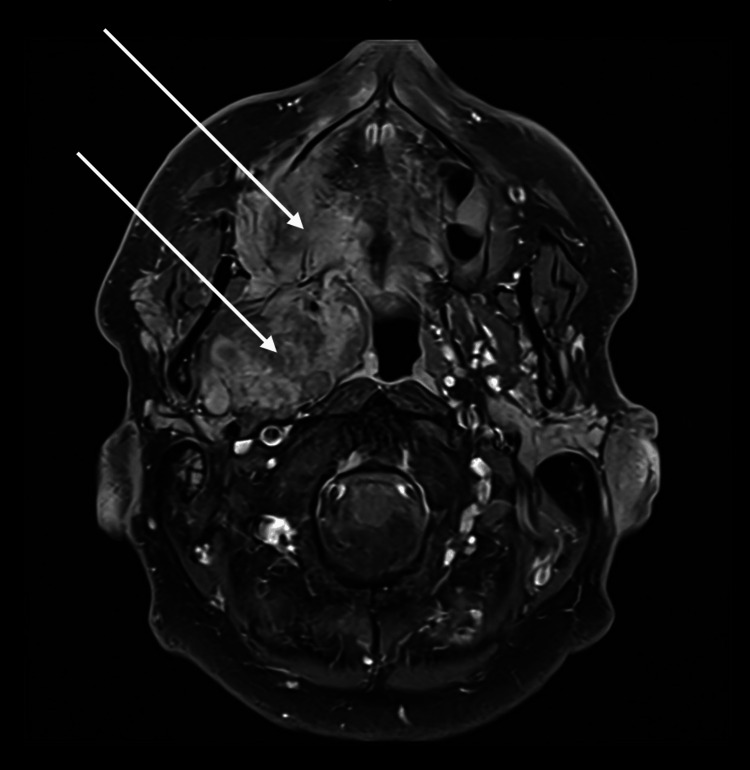
MRI demonstrating a large, locally invasive aggressive maxillary tumour involving the nasopharynx. There is bony destruction with soft tissue extension into the masticator space, pterygopalatine fossa, nasal cavity, and inferior orbital wall (arrows).

Magnetic resonance imaging (MRI) showed a large, locally invasive maxillary tumour involving the floor of the maxillary sinus, with bony destruction and soft tissue extension into the masticator space, pterygopalatine fossa, nasopharynx, and inferior orbital wall (Figure 1).

There was perineural spread and significant lymphadenopathy in the right masticator space. Differential diagnosis included advanced squamous cell carcinoma of the right maxilla or, less likely, primary nasopharyngeal carcinoma. Axial MRI of the head showing a large tumor centered on the right maxilla with local invasion.

**Figure 2:**
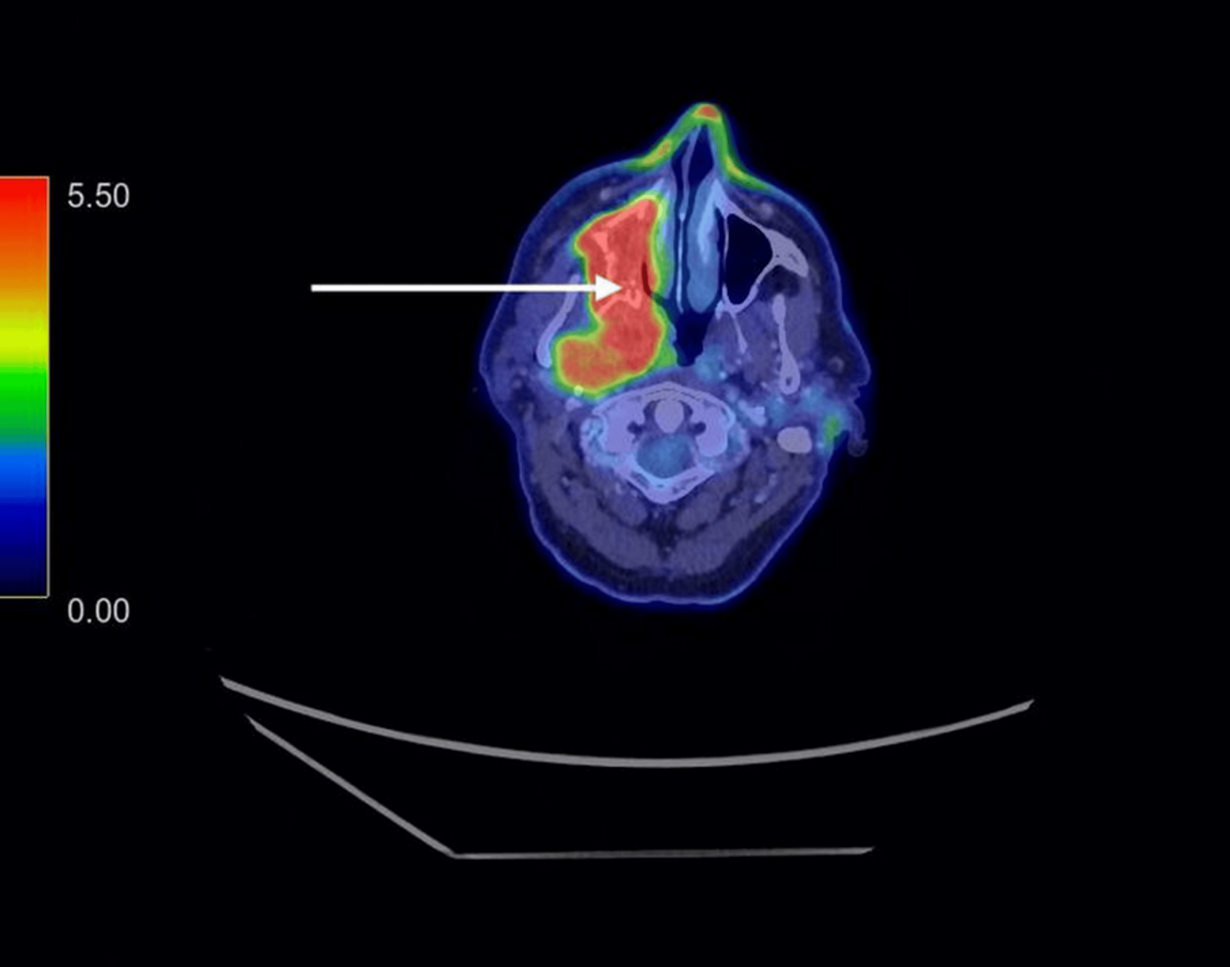
PET-CT showing an extensive right-sided maxillary mass involving the nasopharynx and maxillary sinus, with osseous destruction, pterygoid involvement, and extension into the parapharyngeal space (arrow). PET-CT: positron emission tomography-computed tomography

A positron emission tomography-computed tomography (PET-CT) scan (Figure 2) revealed an extensive right-sided maxilla mass involving the nasopharynx, maxillary sinus with osseous destruction, pterygoid involvement, and extension to the parapharyngeal space. Superiorly, it extended to the foramen ovale and abutted the cavernous sinus. The areas of enhancing tissue demonstrated diffuse fluorodeoxyglucose (FDG) uptake, with a standardized uptake value (SUV) of 11.7. This CT demonstrated marked osseous destruction involving the right maxillary sinus as well as the inferior aspect of the maxilla, involving the maxillary teeth, as well as some destruction of the pterygoid plates, both medial and lateral aspects. Right neck nodes at levels IB and II were involved, alongside bulky mediastinal nodes. Numerous pulmonary, hepatic, and osseous metastases were identified, including at the C7 vertebra.

**Figure 3:**
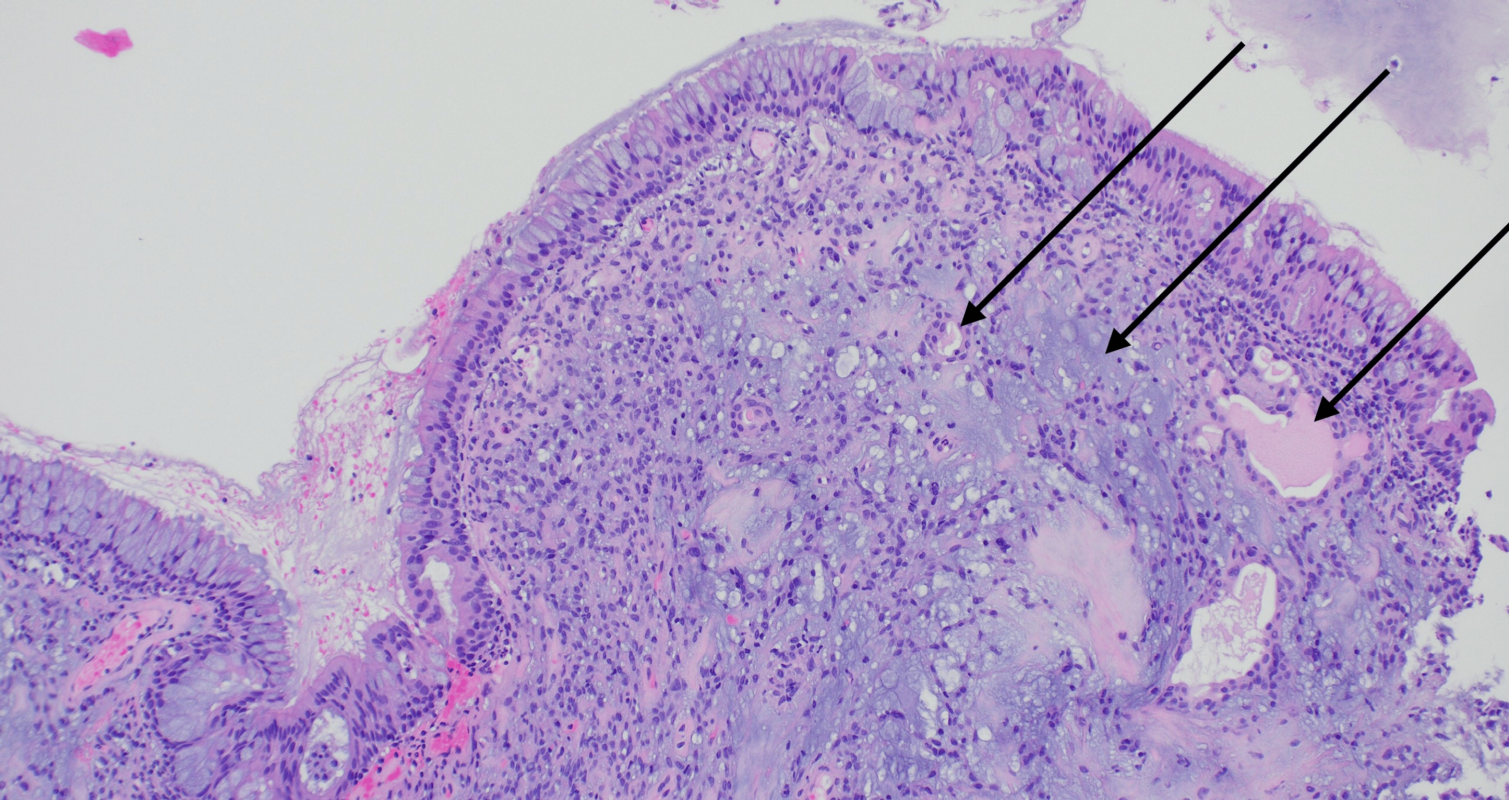
Histology from a nasopharyngeal biopsy demonstrating a pleomorphic adenoma located beneath the respiratory epithelium (arrows; H&E, 100× magnification). H&E: hematoxylin and eosin

**Figure 4:**
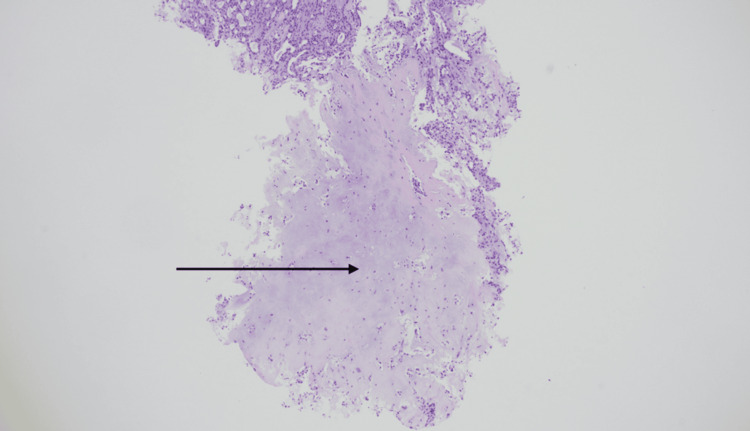
Histology from a nasopharyngeal biopsy showing prominent cartilaginous stroma (arrow; H&E, 40× magnification). H&E: hematoxylin and eosin

Histopathological examination of the nasopharyngeal biopsy revealed fragments of PA with cartilaginous stroma and benign epithelial elements, without atypia (Figures 3-4).

**Figure 5:**
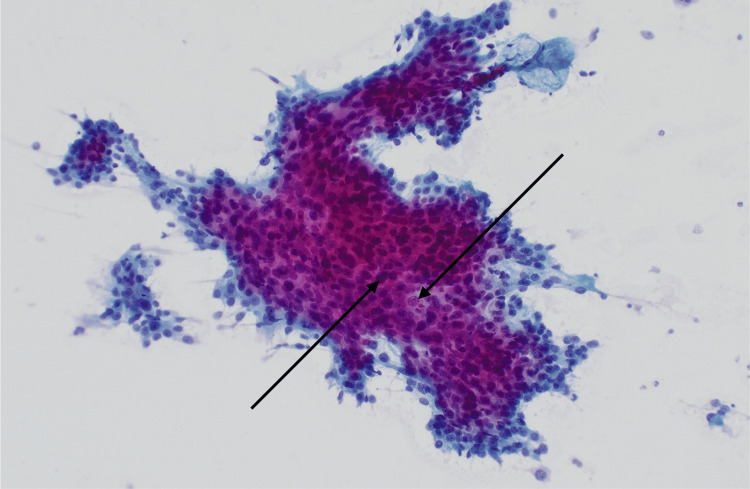
Cervical lymph node fine needle aspiration (FNA) cytology showing sheets of cohesive malignant cells (arrows; PAP stain, 100× magnification). PAP: Papanicolaou

Fine-needle aspiration of a right level II cervical lymph node demonstrated malignant cells with high nuclear-to-cytoplasmic ratios, nuclear irregularities, and macronucleoli (Figure 5).

**Figure 6:**
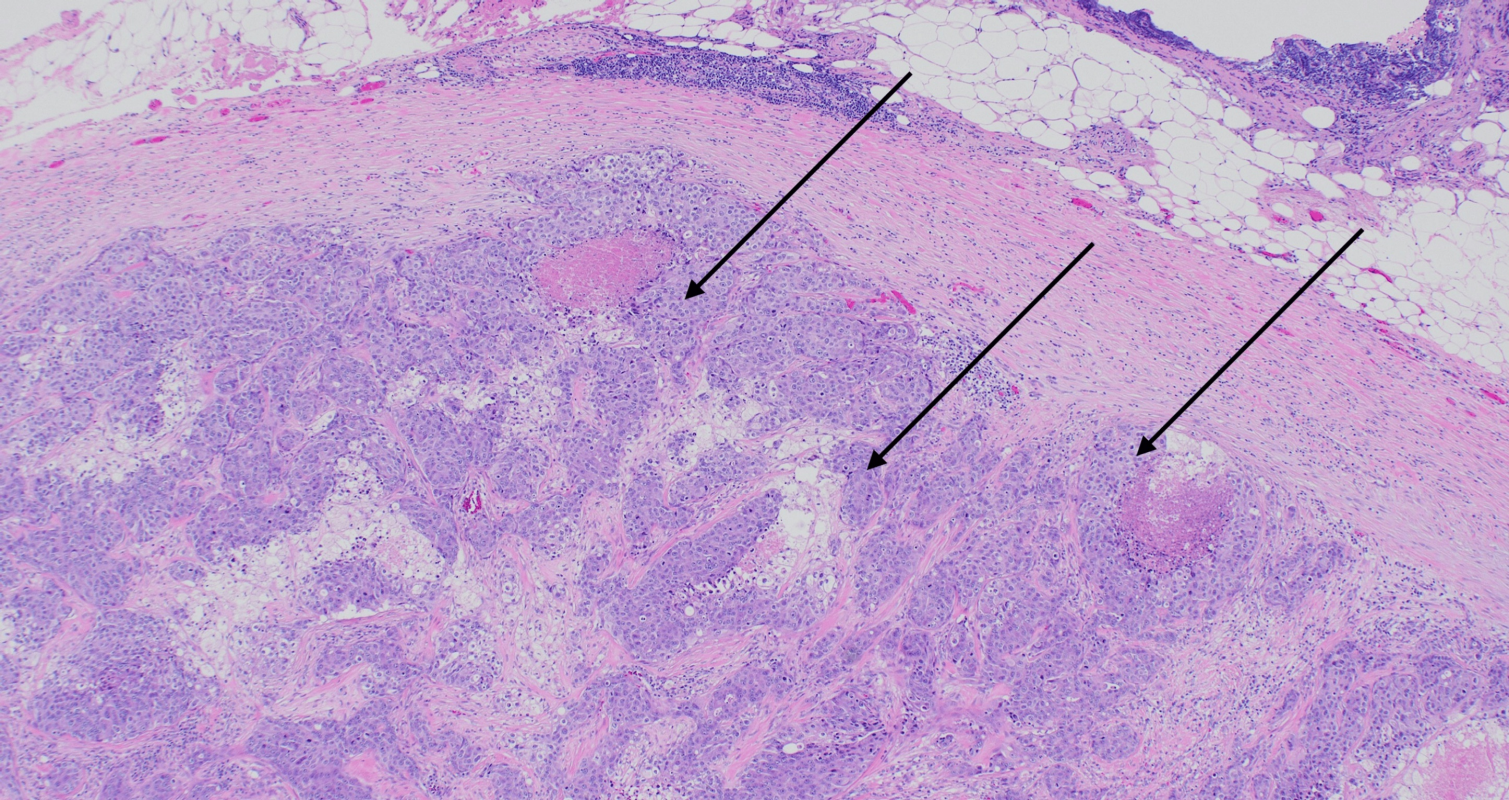
Histology of the cervical lymph node showing replacement by metastatic carcinoma (arrows; H&E, 100× magnification). H&E: hematoxylin and eosin

**Figure 7:**
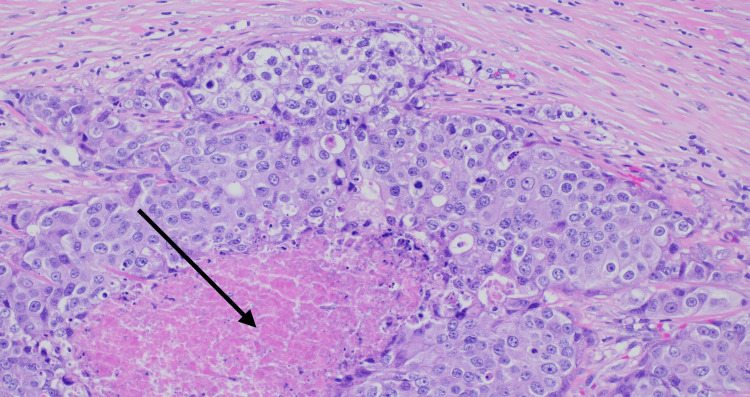
Histology of the cervical lymph node demonstrating metastatic, poorly differentiated neoplasm with comedo necrosis (arrow; H&E, 200× magnification). H&E: hematoxylin and eosin

The lymph node was excised, and histology was performed (Figures 6-7).

**Figure 8:**
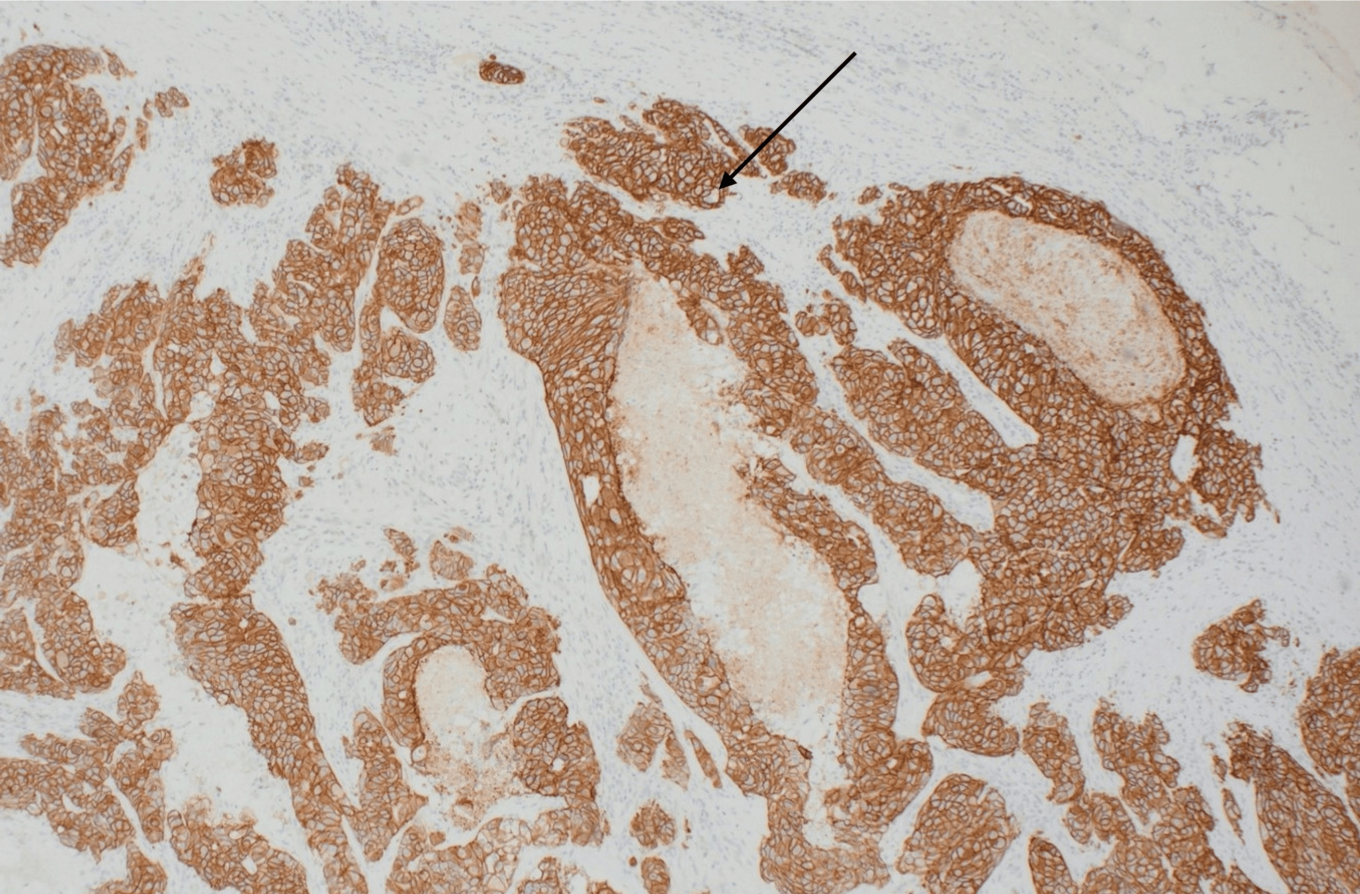
HER2 immunohistochemistry showing strong (3+) membranous staining (arrow).

Immunohistochemistry showed strong membranous positivity of the tumour cells with HER2 (Figure 8) and androgen receptor. P40 and p16 stains were negative. These findings were consistent with metastatic carcinoma, likely CXPA.

**Figure 9:**
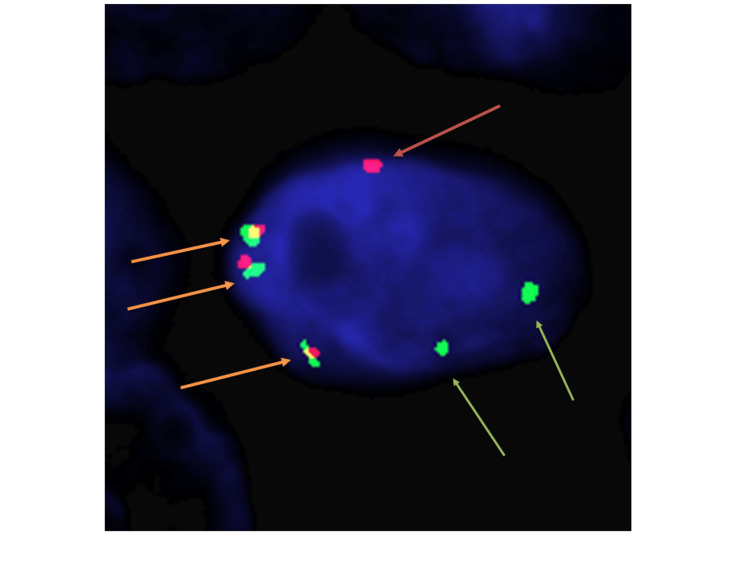
Fluorescence in situ hybridization (FISH). On the left: normal PLAG1 FISH signals (yellow arrows pointing to them) On the right: abnormal break-apart PLAG1 FISH signals (demonstrated by separate red and green signals)

Fluorescence in situ hybridization (FISH) demonstrated PLAG1 gene rearrangements in both the benign nasopharyngeal PA and the metastatic carcinoma in the lymph node, confirming a diagnosis of CXPA (Figure 9).

Upon being informed of the advanced stage of his disease, the patient declined further treatment. His condition deteriorated rapidly, and he died eight weeks after the initial presentation.

## Discussion

CXPA is rare in major salivary glands and extremely uncommon in the nasopharynx. To our knowledge, this is the first reported case of nasopharyngeal PA progressing to CXPA with both nodal and distant metastases. Nasopharynx was established as a primary site based on some radiographic features, and the absence of any other component than PA in the biopsy. The understanding of the full clinical picture was challenging, as the initial biopsy revealed only benign PA, while radiology strongly suggested an aggressive malignancy. This discrepancy prompted molecular analysis, where PLAG1 rearrangement proved essential in confirming the link between the primary PA and metastatic carcinoma. These findings are consistent with previous reports that highlight the difficulty in diagnosing CXPA based solely on biopsy, as malignant transformation can be focal and not captured in small samples [7,8].

The oncogenic role of PLAG1 in PA is well established. Initial cytogenetic studies revealed chromosomal translocations in approximately 70% of PAs [9-12]. Further molecular investigations identified two primary target genes involved in these translocations: PLAG1 [13,14], located at 8q12, in approximately 39% of cases, and HMGA2 [15], located at 12q14-15, in about 8%. The remaining 23% of karyotypically abnormal PAs exhibited a range of sporadic clonal chromosomal aberrations [16]. Our observations confirm that the PLAG1 translocation remains one of the key hallmarks of PA biology and its potential for malignant transformation.

Metastatic CXPA remains poorly understood, with mechanisms likely involving genomic instability and subclonal progression. In this case, rapid dissemination with pulmonary involvement illustrates the potential aggressiveness of nasopharyngeal CXPA.Our findings parallel the aggressive behaviour described in the previously published nasopharyngeal CXPA case report, strengthening the possibility that CXPA arising in this location may exhibit a more aggressive clinical course than its major salivary gland counterparts [17].

HER2 overexpression or gene amplification is implicated in the pathogenesis of several epithelial malignancies, including breast, gastric, and salivary gland carcinomas [18-20]. In CXPA, HER2 dysregulation is increasingly recognized as a driver of malignant transformation through activation of downstream signalling cascades such as PI3K/AKT and MAPK, leading to increased proliferation, survival, and metastatic potential [20]. HER2 gene mutation was likely a contributing factor in this particular case.

CXPA most often has the morphology of salivary duct carcinoma (myoepithelial carcinoma and epithelial myoepithelial carcinoma are less common components); therefore, CXPA and de novo salivary duct carcinoma will look morphologically indistinguishable [21]. They share secondary oncogenic events (HER2 overexpression, androgen receptor positivity) [22], but only CXPA shows the PA-type translocations (PLAG1/HMGA2), inherited from its benign precursor (Table 1) [23].

**Table 1:** Comparison of cytogenetic and immunohistochemical characteristics of CXPA and salivary duct carcinoma. CXPA: carcinoma ex pleomorphic adenoma; PA: pleomorphic adenoma

	CXPA	Salivary duct carcinoma
Origin	From a PA	De novo (no PA precursor)
PLAG1/HMGA2 translocation	Often present (inherited from PA)	Absent
HER2 amplification	Common	Common
TP53, PIK3CA mutations	Common	Common
AR expression	Common	Common
Molecular hallmark	Fusion-driven + secondary oncogenic events	High-grade carcinoma mutations only

Our case reflects that the PLAG1 translocation remains one of the distinguishing features of CXPA, providing a molecular rationale for differential diagnosis.

HER2 overexpression and androgen receptor positivity offer potential modalities for treatment. Unfortunately, in this case, the disease progressed too rapidly for medical oncology to be useful. The patient succumbed to the disease eight weeks after the presentation. Comparison with previous cases suggests that while CXPA shares oncogenic features with salivary duct carcinoma [22], clinical behavior may vary based on tumor location and stage at diagnosis.

The distinctive features of this case are the nasopharyngeal origin of PA, its progression to malignancy with metastases, and the use of FISH to genetically confirm the link between primary and metastatic sites. Whereas most published cases describe a more indolent clinical course [23], our case showed unusually rapid dissemination despite sharing hallmark oncogenic events reported in the literature, such as PLAG1 rearrangement [24] and HER2 dysregulation [20]. This contrast highlights that although the molecular profile overlaps with conventional salivary gland CXPA, tumor behavior may be more aggressive in extra-salivary locations. Overall, this case underscores the importance of correlating imaging, histology, cytology, and molecular studies in rare tumours, as reliance on routine histology alone may be misleading. Ancillary testing, such as FISH, may be critical for accurate diagnosis when routine histology is inconclusive.

## Conclusions

We present the first case in the literature of nasopharyngeal PA transforming to CXPA and producing both regional nodal and distant metastases. We have utilized FISH for PLAG1 rearrangement in establishing the link between the primary site and metastasis, highlighting the diagnostic value of that auxiliary test. This case demonstrates the importance of considering CXPA in differential diagnoses of nasopharyngeal tumours and metastases of unknown origin. Regional metastasis of an apparent nasopharyngeal primary could represent CXPA; therefore, FISH could be used to rule out that possibility. Confirmation of HER2 overexpression and androgen receptor positivity in these aggressive neoplasms reinforces the potential utility of targeted therapies.
